# Schizotypal Perceptual Aberrations of Time: Correlation between Score, Behavior and Brain Activity

**DOI:** 10.1371/journal.pone.0016154

**Published:** 2011-01-18

**Authors:** Shahar Arzy, Christine Mohr, Istvan Molnar-Szakacs, Olaf Blanke

**Affiliations:** 1 Laboratory of Cognitive Neuroscience, Brain Mind Institute, Ecole Polytechnique Fédérale de Lausanne (EPFL), Lausanne, Switzerland; 2 Department of Neurology, Hadassah Hebrew University Hospital, Jerusalem, Israel; 3 Department of Experimental Psychology, University of Bristol, Bristol, United Kingdom; 4 Department of Psychology, University of Lausanne, Lausanne, Switzerland; Cedars-Sinai Medical Center and University of California Los Angeles, Gene Therapeutics Research Institute, United States of America

## Abstract

A fundamental trait of the human self is its continuum experience of space and time. Perceptual aberrations of this spatial and temporal continuity is a major characteristic of schizophrenia spectrum disturbances – including schizophrenia, schizotypal personality disorder and schizotypy. We have previously found the classical Perceptual Aberration Scale (PAS) scores, related to body and space, to be positively correlated with both behavior and temporo-parietal activation in healthy participants performing a task involving self-projection in space. However, not much is known about the relationship between *temporal* perceptual aberration, behavior and brain activity. To this aim, we composed a *temporal* Perceptual Aberration Scale (tPAS) similar to the traditional PAS. Testing on 170 participants suggested similar performance for PAS and tPAS. We then correlated tPAS and PAS scores to participants' performance and neural activity in a task of self-projection in time. tPAS scores correlated positively with reaction times across task conditions, as did PAS scores. Evoked potential mapping and electrical neuroimaging showed self-projection in time to recruit a network of brain regions at the left anterior temporal cortex, right temporo-parietal junction, and occipito-temporal cortex, and duration of activation in this network positively correlated with tPAS and PAS scores. These data demonstrate that schizotypal perceptual aberrations of both time and space, as reflected by tPAS and PAS scores, are positively correlated with performance and brain activation during self-projection in time in healthy individuals along the schizophrenia spectrum.

## Introduction

A fundamental trait of the human self is the continuous mental projection to different points in time in order to re-experience past events and predict future occurrences [1], [2], [3]. This is related on cognitive functions including episodic memory [4], future prediction [5], [6] visual imagery [7] and “mental time travel” [8]. The projection of the self along time allows the self to act as “observer, agent, and guardian of the continuity of experience” [9] (p. 161). Continuity of the self in space is also fundamental for human cognition, and mental “projection” of the self to the so-called “third person perspective” is essential for cognitive functions such as agency, self–other distinction, and mental own-body imagery [10], [11], [12], [13]. The similarity between this magnitudes of space and time with respect to the experiencing self has been recently stressed [14], [15]. Disturbances in this spatial and temporal unity of the self is a major characteristic of schizophrenia spectrum disorders – including schizophrenia, schizotypal personality disorder and schizotypy [16], [17], [18], [19], [20], [21], [22], [23], [24], [25], [26]. Accordingly, high frequency of spontaneously experienced schizotypal perceptual aberrations with respect to body and space, as measured by the Perceptual Aberration Scale (PAS) [27], have been considered an indicator of psychosis-proneness [28], [29], [30]. In view of this, we have previously found PAS scores to be positively correlated with behavior and duration of temporo-parietal activation in participants performing a task involving self-projection in space [25], [31], in agreement with other neuroimaging studies, showing higher temporo-parietal activity during space-related tasks (such as agency or visual perspective taking) [24], [31], [32], [33], [34]. However, not much is known about perceptual aberrations in time in people along the schizophrenia spectrum, as well as the relationship between these “temporal” perceptual aberrations and neural activity [35], [36].

**Figure 1:**
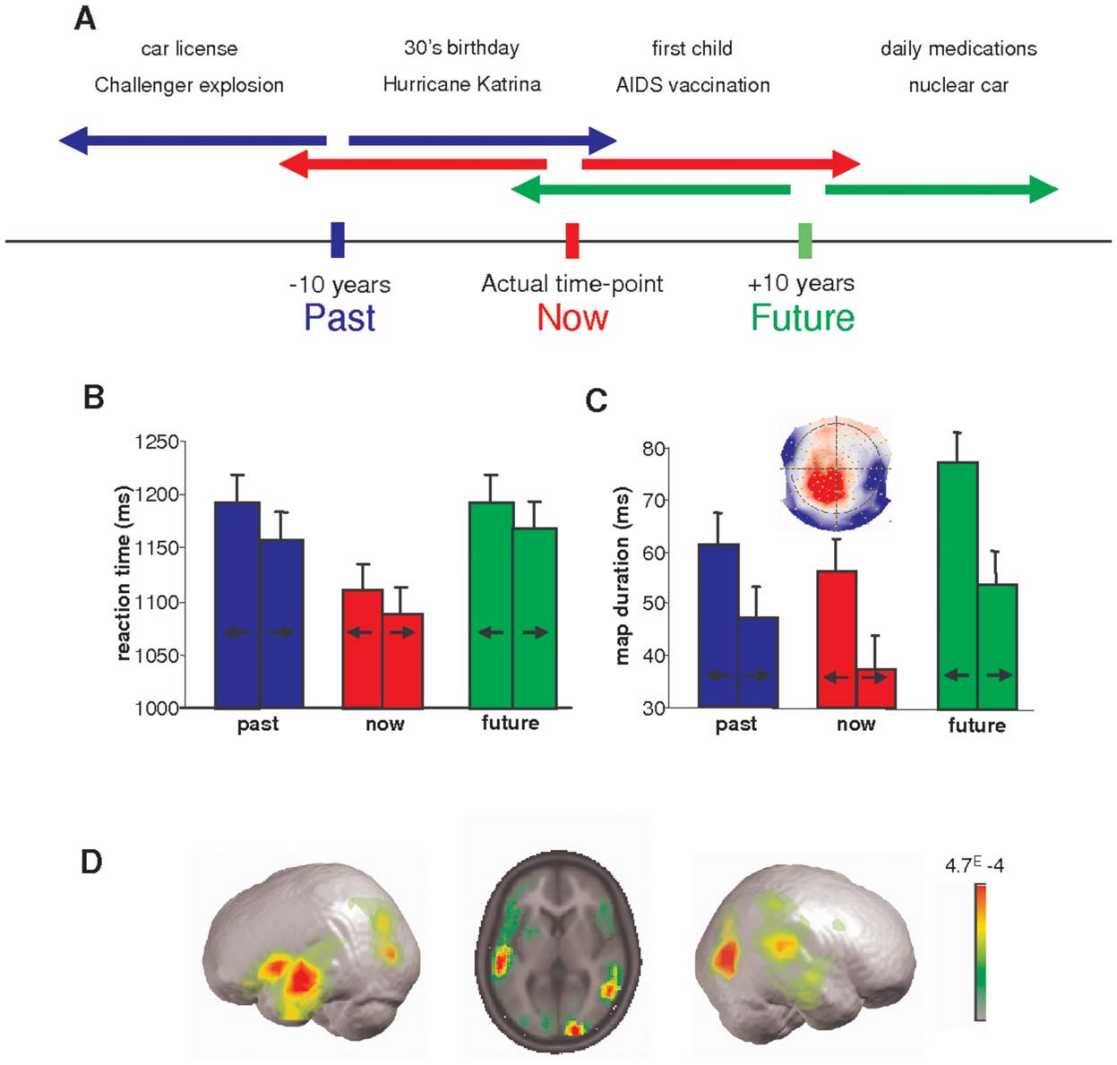
Experimental design and results. (A). Stimuli and procedure. The three different self-projections in time (past, now, and future) are shown. Participants were asked to mentally “project” themselves to one of these time-points, and from these time-points to judge whether different self or nonself events (e.g., top row) already happened (relative past) or are yet to happen (relative future). (B) Behavioral data. Reaction times are plotted separately for past (blue), now (red), and future (green) self-projections in time. Note the significantly higher results for past and future with respect to the now. (C) From all EP maps, only one “time-map” was found to be significantly longer for past and future than for the now. Topography and duration of this map for the three experimental conditions is shown. (D) Generators of the “time-map” were localized to the right temporoparietal, occipitotemporal, and left anterior temporal cortices.

To this aim, we first composed a temporal Perceptual Aberration Scale (tPAS; Table 1) similar to the abovementioned PAS scale [37], [38]. The tPAS was, secondly, tested in a large group of 170 healthy participants. We thirdly compared tPAS and PAS scores to participants' performance in a smaller group of 14 participants performing a self-projection in time task, as reported previously [3], and fourthly, compared tPAS and PAS scores to participants' brain activity as measured by electrical neuroimaging during task performance. The task asked participants to imagine themselves at three different time-points: Now (the present time-point), Past (10 years earlier than the present time-point), or Future (10 years later). In separate blocks for the Now, Past, and Future time-points, participants were shown a series of different events (personal (e.g. first child) or non-personal (e.g. Obama's election) on a computer screen. They were asked to indicate if the presented event took place before (Backwards in time) or after (Forward in time) the currently imagined time-point. Accordingly, we labelled this latter behaviour relative self projection, whereas the projection to past, now or future points was labelled absolute self projection (Figure 1A). Regarding our previous results showing positive correlation between PAS scores and behaviour and brain activity in self-projection in space, as well as the similarity between self projection in time and space [1], [3], [12], [15], [23], [31], we hypothesized that performance and brain activity in this self-projection in time task will be correlated with level of spontaneously experienced schizotypal perceptual aberrations in space and time as measured by PAS and tPAS scores.

**Table 1:** Temporal Perceptual Aberrations Scale (tPAS).

*Statement*
1. I sometimes have the feeling that I have already been in a situation like the one I am currently in (déjà vécu).
2. I sometimes re-imagine experiences I had in the past.
3. I dedicate much time to planning my schedule.
4. I sometimes have the feeling of having already seen things that are presented to me (déjà vu).
5. I sometimes find myself dreaming awake.
6. I sometimes look upon myself from a future point of view.
7. I sometimes imagine myself as a younger child.
8. I sometimes have a dream in which I meet my past self.
9. I sometimes have a dream in which I meet my future self.
10. I sometimes think deeply about my old age.
11. I sometimes know what to do as I already predicted such a situation without previously experiencing it.
12. I sometimes feel that I am older than my current age.
13. I sometimes feel that I am younger than my current age.
14. I sometimes have the feeling that I know what is going to happen.
15. I sometimes regret key decisions I took in my life.
16. I am sometimes bothered by key decisions I have to make in the far future.
17. I sometimes believe that patterns or situations that have already happened will re-occur again.
18. I am sometimes not sure if some events had really occurred to me or were just imagined.
19. I sometimes feel sure when performing an action, although I have never done it before.
**20. I am sometimes not sure if I did something that I had already done.**

The 20-item tPAS scale, which was developed for measuring the frequency of schizotypal perceptual aberrations of time, is presented. Participants had to rate on a scale from 1 to 10 how much these statements are true with respect to themselves, based on their own experiences (1 - not at all; 10 – very much).

## Results

### Behavioural testing of the temporal Perceptual Aberration Scale (tPAS)

Analysis of results obtained from the 170 participants for the whole sample showed a significant positive correlation between PAS and tPAS scores (r = 0.57, p<0.001, two-tailed). This suggests that participants performed similarly in the PAS and tPAS. The same correlation conducted for women and men separately showed this correlation to be evident for each sex (men: r = 0.54, p<0.001; women: r = 0.60, p<0.001). Independent t-tests showed that neither the mean tPAS score, nor the mean PAS score differed (tPAS: t_168_ = 1.10, p = 0.27, PAS: t_168_ = 1.65, p = 0.10) between women (tPAS: 4.55±1.27, PAS: 6.49±5.58) and men (tPAS: 4.35±1.19, PAS: 5.29±3.75).

### tPAS and PAS Questionnaires

Mean tPAS score for the electrical neuroimaging group was 4.0±1.3. PAS score of 5.5±2.1 was comparable to those reported in previous studies [25], [27], [31], [39]. tPAS and PAS scores were found to be correlated to each other (r = 0.72, p<0.01).

### Task performance and questionnaire scores

**Figure 2:**
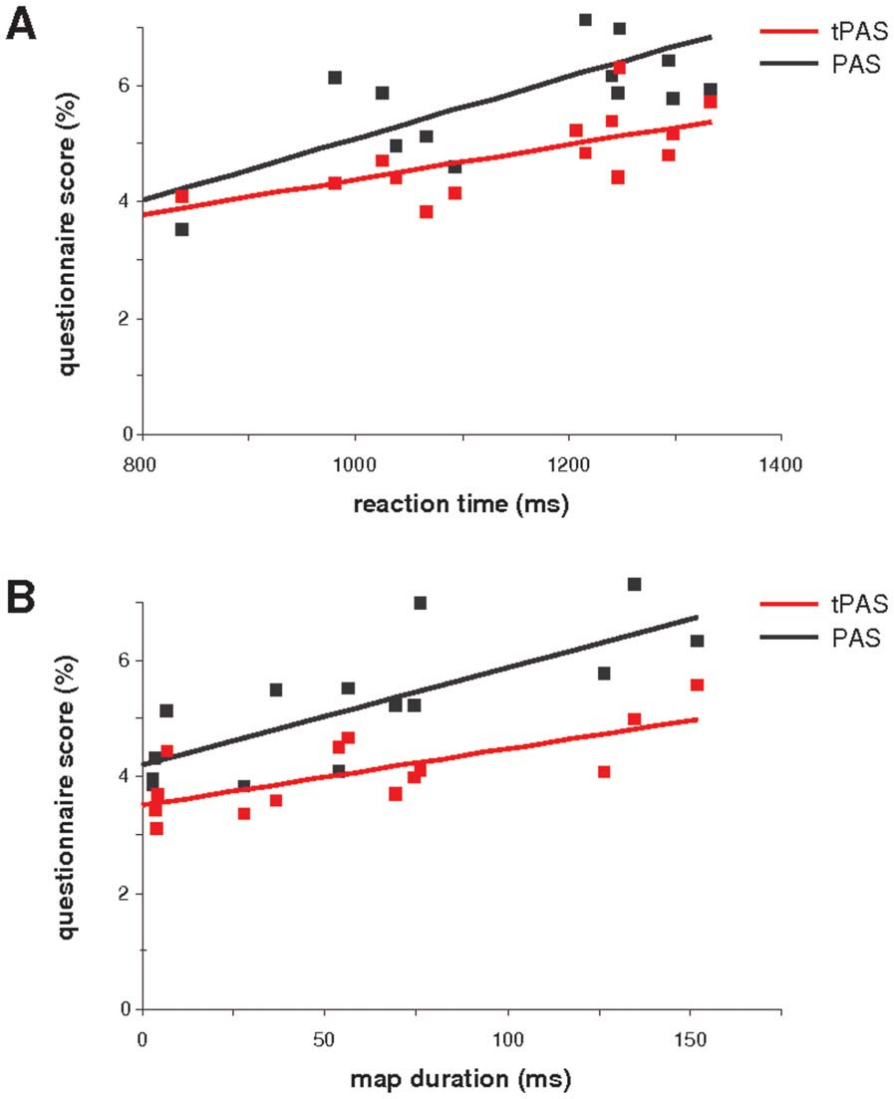
Correlations between reaction times, brain activation, and temporal and spatial perceptual aberration scale (tPAS, PAS) scores. (A) tPAS and PAS scores as a function of reaction times in the self-in-time task. (B) tPAS and PAS scores as a function of duration of activation of the time-map as measured by electrical neuroimaging. Note that this correlation parallels the correlation between the behavioral results and the tPAS and PAS scores.

Reaction times in the time task were significantly longer for Past (mean±SD: 475.3±137.1 ms) and Future (480.1±154.1 ms) than Now (401.1±159.2 ms) events (F_(2,26)_ = 12.5 p<0.001; Figure 1B) [3], [40]. tPAS scores correlated positively with reaction times across task conditions (r = 0.69, p<0.01), as did the PAS scores (r = 0.68, p<0.01; Figure 2A). With regard to accuracy, participants' error rates were higher in the Past and Future conditions than in the Now condition (mean error rates: 9.2±5.2% (Past); 6.1±3.1% (Now); 9.8±4.5% (Future); F_(2,26)_ = 7.2, p<0.01). Accuracy rates did not correlate with either tPAS scores (r = 0.16, p = 0.54) or with PAS scores (r = 0.09, p = 0.79).

### EP mapping and source localization

EP mapping of the group-averaged data revealed one microstate of brain activation (time segment of stable voltage topography; EP map) that lasted significantly longer for the Past (110.2±12.3 ms) and Future (105.0±15.2 ms) conditions than the Now (39.2±8.4 ms) condition as reported previously (F_(2,26)_ = 3.8, p = 0.03; Figure 1C) [3], comparable to the behavioural data pattern, suggesting that this EP map is linked to self-projection in time (time-map). No difference was found when statistical analysis was performed on the amplitude (or GFP) of those EP maps (F_(2,26)_ = 0.6; *p* = 0.56). A linear inverse solution (LAURA) [41] localized the time-map to the left anterior temporal cortex, right temporo-parietal junction, and occipito-temporal cortex (Figure 1D) [40].

### Questionnaire scores and neural activation

Correlation analysis of tPAS and PAS scores with the duration of the time-map for each subject revealed significant positive correlations (tPAS: r = 0.59, p<0.05; PAS: r = 0.58, p<0.05; Figure 2B), comparable to the relationship between behavioral reaction times and tPAS and PAS scores. Neither tPAS nor PAS scores were significantly correlated with the strength (or GFP) of the time-map (tPAS: r = 0.44, p = 0.11; PAS: r = 0.43, p = 0.12). The duration or the amplitude of no other EP map, before and after the time-map, showed a significant correlation with these scores. These data suggest that during the self-projection in time task, the duration, but not amplitude (GFP) of neural activation, in a specific time period (∼350–500 ms after stimulus onset), within a network including left anterior temporal cortex, right temporo-parietal junction, and occipito-temporal cortex, is positively correlated with schizotypal symptomatology, specifically temporal and spatial perceptual aberrations as measured by tPAS and PAS scores, respectively. These data suggest that schizotypal perceptual aberrations of time and space are positively correlated with behavioral performance and neural activation during a self-projection in time task.

## Discussion

### Time, space and schizotypy

Impairment of self- and spatial-processing is a prominent characteristic of disorders along the schizophrenia spectrum [17], [19], [21], [22], [24], [26], [27], [33], [42], [43], [44], [45]. However, although similar impairments might be related to the sense of a unified, coherent self over time [23], such temporal aspects of self-processing have received little empirical attention in schizophrenia. Specifically, while several studies investigated temporal processing in the seconds or minutes range [46], [47], [48], [49], [50], [51], almost no attention was paid to longer time periods. However, disturbances of time consciousness in schizophrenia are not restricted to short time scales, but appear to also involve binding together experiences from one's personal history over a time scale of years [52], often referred to as ‘autonoetic awareness’ [8], [52], [53]. In a study to test the hypothesis that patients with schizophrenia are unable to link the separate aspects of events into a cohesive, memorable, and distinctive whole, it was shown that such patients exhibited an impaired recognition memory and a reduction in frequency of autonoetic awareness relative to a control group [54]. A more recent study found that patients with positive symptoms of schizophrenia recalled fewer specific past events than did healthy controls and were even more impaired in generating specific future events [36]. Although not directly tested, these authors speculated that such schizophrenic patients' failures to process past and future episodes might be related to disturbance of the sense of “subjective time” in these patients, as well as to difficulties in episodic memory, which has been assumed to be disturbed in schizophrenia [55], [56], [57], [58], [59]. The present data demonstrate that performance and brain activation in a task of self-in-time positively correlated not only with the degree of spatial perceptual aberrations as reflected by PAS scores [25], [31], but also with *temporal* perceptual aberrations as reflected by tPAS scores. These findings are in line with recent proposals that self-projection in space and time share similar brain mechanisms [1], [3], [15].

Our EP data also showed that participants with higher tPAS and PAS scores took longer to respond in a task involving self-projection in time, and activated the right temporo-parietal junction, the left anterior temporal lobe and the occipito-temporal cortex bilaterally for a longer time. This is also in agreement with studies of patients with schizophrenia revealing impairment in temporo-parietal junction activity related to self-processing [16], [34], [60], [61]. Moreover, this activity was found to be positively correlated with Schneiderian scores of schizophrenia [24], [33]. However, whereas earlier studies suggested that pathological temporo-parietal junction activity was due to changes in strength of activation, the present data suggest that increased temporo-parietal junction activity was due to longer, but not stronger temporo-parietal junction activation [31].

The current findings may be explained by several neural mechanisms that have been put forward to account for prolonged brain activation patterns. David et al. [62] proposed that increases in duration of activation may depend on the increased contribution of top–down connections, reflecting re-entry of neural signals to lower-level processing areas, as EPs were found to be more enduring and dispersed in higher level areas [62]. This is particularly true with respect to late EP components [62], like the time-map that we found for the time period between 350 and 500 ms after stimulus presentation. The prolonged activity found in the present study might thus reflect atypical processing in a network of brain regions at the anterior temporal, temporo-parietal and occipito-temporal cortex related to altered top–down signals in subjects with high schizotypy scores. Alternatively, prolonged activation at these regions may also be due to an increase of independent simultaneous brain processes and/or degraded functional connectivity between these regions [63], [64], [65].

### Schizotypal perceptual aberrations of space and time and their neural mechanisms

Our data suggest that the relative impairment in self-projection in time in healthy participants scoring high on positive schizotypy may be related to atypical processing within the described network. It has been proposed that brain function might be broadly divided into an ‘extrinsic’ system, processing stimuli delivered from the external environment, and ‘intrinsic’ system, related to mental processing of one's body and self [26], [66], [67], [68], [69], [70], [71]. This latter system is overlapping with the neural network found here for self-projection in time [66], [72]. It was hypothesized that a major role of the intrinsic system is simulation of one's probable future, through episodic thinking and comparison of past memories and future predictions [8], [68], [73], [74], [75]. Research on schizophrenia and schizotypy has found as well aberrations in processing of past memories and future predictions: patients with schizophrenia have been shown to exhibit impaired recognition memory and a reduction in frequency of autonoetic awareness suggesting an impairment in linking the separate aspects of events into a cohesive, memorable, and distinctive processing of subjective time in schizophrenia [54]. The current data refine these findings further by showing significant and coherent variability in self-projection in time to past and future in individuals along the schizophrenia spectrum.

In conclusion, the present study demonstrates that self-projection in time as reflected by behavioral measures as well as duration of activation in a network of brain regions including left anteroior temporal, the right temporo-parietal and bilateral occipito-temporal cortex, are positively correlated with degree of schizotypy, as measured by tPAS and PAS scores. These results suggest that individuals with increased levels of schizotypy are relatively impaired in self-relevant processing of space and time.

## Materials and Methods

### Questionnaires

Participants completed the 20-item tPAS scale (Table 1), which was developed for measuring the frequency of schizotypal perceptual aberrations of time. Typical items of the tPAS scale are “I sometimes re-imagine experiences I had in the past” and “I sometimes have the feeling that I know what is going to happen”. Participants had to rate on a scale from 1 to 10 how much these statements are true with respect to themselves, based on their own experiences (1 – not at all; 10 – very much). In addition, they completed the 35-item true–false self-report PAS [27]. Typical items of the PAS are “Occasionally I have felt as though my body did not exist” and “Occasionally I have the impression that one of my body-parts is bigger than usual” [28], [29], [30]. Participants also completed the standardized handedness questionnaire [76] and were asked about their previous neurological or psychiatric history.

### Participants

Behavioural study: 170 healthy volunteers (85 women) aged 20.82±4.62 years (mean±sd; range 18–47 years) were asked to complete the tPAS (Table 1) and PAS questionnaire [27]. Half of the participants were undergraduate students receiving course credit for their participation, while the remaining participants were undergraduate students from various faculties at the University of Bristol, UK. The affiliation of the latter participants remained anonymous, since each undergraduate student of the local psychology department was only asked to recruit an additional undergraduate student of the opposite sex in order to balance gender differences (70% of the psychology students were women, see also [25] for a comparable test setting). Prior to the experiment, which was approved by the local Ethical Committee of the University of Bristol, all participants provided written informed consent. Testing took place in groups of 5–10 in the classroom.

Electrical neuroimaging study: Fourteen healthy volunteers (seven males, aged 29–38 years; 31.5±2.9 years) participated in the behavioural and EEG experiment, as reported previously [3]. All participants were right handed, and had normal or corrected to normal vision and no history of neurological or psychiatric disorders. All participants gave written informed consent before inclusion in the study, which was approved by the Ethical Committee of the University Hospital of Geneva (Switzerland).

### Paradigm

The present study is based on a new analysis of data previously collected and published [3]. Only the most relevant description of paradigm and key results is provided here. Participants were asked to “project” themselves to three different time-points: now (the present time), past (10 years in the past), or future (10 years in the future). In separate blocks for the past, now, and future, two-word phrases describing different common events from personal life (e.g., driver's license; first child) or non-personal world events (e.g., Challenger explosion; Obama's elections) were presented on a computer screen (Figure 1A). Participants were asked to indicate whether the presented event took place before (relative past) or after (relative future) the currently imagined time-point (Figure 1A). Stimuli were designed to be in the range of ±15 years of the imagined time-point. Judgments were given using index and middle fingers of the left and right hand in alternating blocks as a button press on a serial response box. Participants were instructed to respond as quickly and precisely as possible while maintaining a mental image of themselves in the appropriate time-point (past, now, or future), which were performed in six blocks (each repeated once) and counterbalanced across subjects. Each block included 120 stimuli, equally distributed among four groups: self (personal events) in relative past, self in relative future, non-self (world events) in relative past, and non-self in relative future, appearing in random order.

### Analysis of behavioral data

Repeated measures ANOVAs were run on reaction times and accuracy with Time (Past, Now and Future) as the repeated measures factor. Then, to test possible relationships between individuals' questionnaire scores and task performance, separate correlation analyses were performed between participants' reaction times and accuracy results and their questionnaire scores on tPAS and PAS. These were performed for the whole sample, using Pearson product moment correlations. All p-values are two-tailed, and the significance level was set to α = 0.05.

### Electroencephalography (EEG) recording and evoked potential (EP) mapping

Continuous EEG was acquired with a Bio-Semi system (Bio-semi, Inc., Netherlands) from 192 scalp electrodes (impedances <5 kΩ; vertex referenced; 2048 Hz digitization; band-pass filtered 0.1–100 Hz) in a darkened, electrically shielded booth. Epochs of EEG (from 0 to 800 ms post-stimulus onset) from trials yielding correct responses were averaged for each of the three experimental conditions (Past, Now and Future) and for each subject to calculate the EPs [77]. In addition to the rejection of sweeps where any channel exceeded the amplitude of ±100 µV, the data were visually inspected to reject epochs with blinks, eye movements, or other sources of transient noise. EPs were band-pass filtered (1–40 Hz) and recalculated against the average reference [77]. The 192-channel EP analysis was based on the examination of the spatial variations of the voltage distribution over time and between conditions, an approach known as microstates EP mapping [77], [78], [79], [80], [81], [82]. This approach searches for time segments of stable map topography that represent functional microstates of the brain during information processing. EP microstate segments were defined by using a spatial k-means cluster analysis to identify the dominant map topographies in the group-averaged evoked potentials across the experimental conditions over time. The optimal number of these template maps is determined by a modified cross-validation criterion [78], [79], [80], [83]. In a second step the presence of a given EP map as identified in the group-averaged data was verified statistically in the EPs of the individual subjects. EP maps were competitively fitted to the EPs of the individual subjects. This allows determination of the duration (number of time-points that were assigned to one microstate map) and the amplitude (or global field power, GFP) of a given EP map for each condition across subjects. These duration and GFP values for a given EP map then can be subjected to statistical analysis. Statistical comparisons were performed on the duration and GFP of each map (dependent variable) in the individual EPs using repeated measures ANOVAs, with Time as the repeated measures factor. Then, to test possible relationships between individuals' questionnaire scores and the measured brain activity, the respective mean durations and GFP of the different EP maps for the individual subjects were correlated with participants' tPAS and PAS scores. For each EP map, separate correlation analyses were performed (for duration and GFP) for both questionnaire scores. These were performed for the whole sample, using Pearson product moment correlations. All p-values are two-tailed, and the significance level was set to α = 0.05.

### Source localization

The neural generators for a given mean EP map were estimated by using a distributed linear inverse solution, based on a local auto-regressive average (LAURA) model [41]. LAURA selects the source configuration that tries to mimic the biophysical behavior of electric vector fields (i.e., activity at one point depends on the activity at neighboring points according to electromagnetic laws). The solution space was calculated on a realistic head model that included 4024 nodes, selected from a 6×6×6 mm-grid equally distributed within the gray matter of the Montreal Neurological Institute's average brain.
